# Acoustic Radiation Force Impulse Imaging: A New Tool for the Diagnosis of Papillary Thyroid Microcarcinoma

**DOI:** 10.1155/2014/416969

**Published:** 2014-06-22

**Authors:** Yi-Feng Zhang, Chang Liu, Hui-Xiong Xu, Jun-Mei Xu, Jing Zhang, Le-Hang Guo, Shu-Guang Zheng, Lin-Na Liu, Xiao-Hong Xu

**Affiliations:** ^1^Department of Medical Ultrasound, Shanghai Tenth People's Hospital, Tenth People's Hospital of Tongji University, No. 301 Yanchangzhong, Road Shanghai 200072, China; ^2^Department of Ultrasound, Guangdong Medical College Affiliated Hospital, Zhanjiang 524001, China

## Abstract

*Purpose*. To evaluate the diagnostic performance of ARFI imaging in differentiating between benign and malignant thyroid nodules <1 cm. *Materials and Methods*. 173 pathologically proven thyroid nodules (77 benign, 96 malignant) in 157 patients were included in this study. Receiver-operating characteristic curve (ROC) analyses were performed to assess the diagnostic performance of conventional ultrasound (US) and ARFI imaging in papillary thyroid microcarcinoma (PTMC). The independent risk factors for predicting PTMC were evaluated. *Results*. The mean SWV value of benign and malignant thyroid nodules were 2.57 ± 0.79 m/s (range: 0.90–4.92 m/s) and 3.88 ± 2.24 m/s (range: 1.49–9.00 m/s) (*P* = 0.000). Az for VTI elastography score was higher than that for hypoechoic, absence of halo sign, and type III vascularity (*P* < 0.05). The optimal cut-offs for VTI elastography score and SWV were score 4 and 3.10 m/s. Gender, hypoechoic, taller than wide, VTI elastography score ≥ 4, and SWV > 3.10 m/s had been found to be independent risk factors for predicting PTMC. *Conclusion*. ARFI elastography can provide elasticity information of PTMC quantitatively (VTQ) and directly reflects the overall elastic properties (VTI). Gender, hypoechogenicity, taller than wide, VTI elastography score ≥ 4, and SWV > 3.10 m/s are independent risk factors for predicting PTMC. ARFI elastography seems to be a new tool for the diagnosis of PTMC.

## 1. Introduction

The incidence of papillary thyroid cancer (PTC) is increasing around the world according to several epidemiological studies. The increase is mainly due to the detection of papillary carcinomas less than 1-2 cm in size, while the incidence of larger tumors is stable [1–3]. The thyroid carcinoma can be defined as a thyroid microcarcinoma (TMC) when it is less than 10 mm in size. Since the vast majority of thyroid cancers are PTC, which accounts for more than 80% of all thyroid malignancies [1–4], the preferred definition is now micropapillary thyroid carcinoma (mPTC). The pathological description which best defines mPTC is “a tumor ranging from 0.1 to 1.5 cm (mostly between 0.4–0.7 cm), microscopically nonencapsulated, sclerotic, often located subcapsularly, mainly papillary, often infiltrating the surrounding thyroid” [5]. Most papillary thyroid microcarcinomas (PTMCs) have an excellent prognosis; however, there are some PTMCs which have a grave course showing recurrence and distant metastasis and leading to mortality according to several recent studies [6, 7]. The guidelines of the American Thyroid Association (ATA) recommended that only nodules >1 cm should be evaluated, since they have a greater potential to be clinically significant cancers and nodules <1 cm only required fine-needle aspiration (FNA) because of suspicious ultrasound (US) findings, associated lymphadenopathy, a history of head and neck irradiation, or a history of thyroid cancer in one or more first-degree relatives [8]. Furthermore, FNA has limitations in small thyroid nodules because of its high inadequate specimen rate. The diagnosis of PTMCs was relatively difficult compared with the carcinoma >1 cm. Some recent studies showed that acoustic radiation force impulse (ARFI) elastography was useful for the differentiation between benign and malignant thyroid nodules [9–11]. However, there is no study on the usefulness of ARFI on the diagnosis of PTMC till now. The aim of this study was to assess the usefulness of ARFI in the differential diagnosis between benign and malignant thyroid nodules ≤1 cm.

## 2. Materials and Methods 

### 2.1. Patients

**Figure 1:**
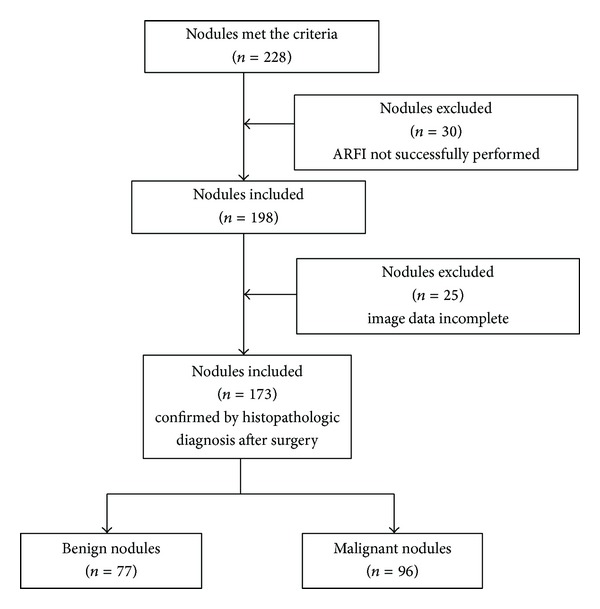
The flowchart of selection of the patients with thyroid nodules.

From July, 2011, to July, 2013, 173 nodules in 157 patients were included in this retrospective study. The inclusion criteria of the target nodules were as follows: (1) suspicions of malignancy based on the features on gray-scale US; (2) the diameter of the nodules ranged from 0.5 cm to 1.0 cm; (3) solid or almost solid (<25% cystic) nodules; (4) the cystic and calcified portions of the nodule can be excluded out of the region of interest (ROI) when using the ARFI examination; (5) no treatment such as ablation or biopsy was performed on the nodules; (6) enough thyroid tissue surrounding the nodule at the same depth; (7) pathological results were obtained. The flowchart for the selection of thyroid nodules was shown in Figure 1. All the nodules were confirmed by histopathological diagnosis after surgery. The indications for thyroidectomy were as follows: (1) confirmed malignancy by FNA; (2) highly suspicious of malignancy by US or FNA; (3) Compressive symptoms caused by the associated large nodules. They were 122 female and 35 male patients. The mean age of these patients was 50 ± 10 years (range, 22 to 78 years). The diameter of the nodules ranged from 0.5 cm to 1.0 cm (mean, 0.78 ± 0.15 cm). There were 31 patients with single nodule and 126 patients with multiple nodules. For the patients with multiple nodules, the nodules that were suspicious of malignancy (based on conventional US findings) or the largest solid ones were selected for analysis. Informed consent was obtained from all patients, and the local Ethics Committee approved the study.

### 2.2. Conventional US Imaging

All gray-scale US and color Doppler flow imaging (CDFI) images were obtained by using the same scanner (S2000 US machine, Siemens Medical Solutions, Mountain View, CA, USA) and same linear array transducer (9L4, Siemens Medical Solutions, Mountain View, CA, USA) with a center frequency of 7.5 MHz (range, 5.0–14.0 MHz).

The conventional US and ARFI elastography imaging were performed by one investigator who had more than 5-year experience in thyroid US. On gray-scale US, the following features of the nodules were evaluated: echogenicity, margin, calcification, shape, and halo sign. Echogenicity was classified as hyper-, iso-, hypoechogenicity (in comparison with the normal thyroid tissue) or marked hypoechogenicity (when a nodule showed relatively hypoechogenicity compared with the surrounding strap muscle). Margin was classified as well-defined or ill-defined (microlobulated or irregular margin). Shape was classified as ovoid to round, taller than wide, or irregular. Calcification was classified as microcalcification (less than or equal to 1 mm in diameter; tiny, punctate, hyperechoic foci, either with or without acoustic shadows), macrocalcification (including calcification more than 1 mm in diameter and eggshell calcification), or no calcification. Halo sign was classified as presence or absence of halo sign. The color Doppler flow pattern was classified into three types: type I, absence of blood flow; type II, perinodular and absent or slight intranodular blood flow; type III, marked intranodular and absent or slight perinodular blood flow.

### 2.3. ARFI Elastography

ARFI elastography images were obtained after evaluating the nodules with conventional US using the same probe and by the same operator. ARFI elastography included virtual touch tissue quantification (VTQ) and virtual touch tissue imaging (VTI). The patient was asked to hold the breath when the VTI and VTQ mode was initiated. The probe was placed gently on the body surface with light pressure to the thyroid. VTI and VTQ imaging were performed both on the long axis dimension of the nodule.

#### 2.3.1. VTI Elastography Imaging

VTI of ARFI was then carried out firstly. The VTI image reflects the elasticity of tissue with gray-scale image in the field of view (FOV), in which the dark indicates hard tissue whereas the bright indicates soft tissue. The FOV is adjusted to include the whole lesion (approximately occupying 70% of the whole FOV) and some surrounding thyroid tissue (approximately occupying 20–30% of the whole FOV). The VTI images of the thyroid lesions were thereafter scored according to Xu's scoring system [9]: VTI elasticity score (VES) 1, the nodule is displayed predominantly in bright (dark portion, 0–20%); VES 2, the nodule is displayed predominantly in bright with few dark portion (dark portion, 20–40%); VES 3, the nodule is displayed equally in dark and bright (dark portion, 40–60%); VES 4, the nodule is displayed predominantly in dark with a few bright spots (dark portion, 60–80%); VES 5, the nodule is displayed almost completely in dark (dark portion, >80%); VES 6, the nodule is displayed completely in dark without bright spots (dark portion, 100%).

#### 2.3.2. VTQ Elastography Imaging

After the VTI imaging, VTQ was performed. VTQ can reflect the elasticity of tissue quantitatively with the shear wave velocity (SWV). The ROI was placed on the solid portion of the nodule. The calcified and liquefaction necrosis portions of the nodule were avoided during measurement. The size of ROI was 6 mm × 5 mm and cannot be altered. The SWV value was displayed on the screen as m/s (range from 0 to 9 m/s). If the stiffness of the tissue is beyond the limits of measurement, whether high or low, the SWV would be displayed as “x.xx m/s.” The measurement was repeated for 7 times. The median of all 7 measurements per lesion was calculated and used for further analysis. The value of “x.xx m/s” was allocated to be 9 m/s after excluding the possible influencing factors such as patient's respiration or motion and operator's inappropriate gesture [12], because all the nodules included in the study were solid or almost solid; thus, “x.xx m/s” was not allocated to be 0 m/s (i.e., corresponding cystic portion).

### 2.4. Statistical Analysis

Statistical analysis was performed by using a software package (SPSS, version 17.0 for Windows; SPSS, Chicago, Ill). Quantitative data were expressed as mean ± standard deviation. The mean ages and mean diameters of benign and malignant nodules were compared with* t*-statistics. The ratios of male/female, single nodule/multiple nodules, size and location distribution, and other gray-scale US features were compared with chi-square statistics. If there were cells that had less than 5 observations, Fisher's exact probability test was used. The pathology types and VTI scores of benign and malignant nodules were also compared with chi-square statistics. The sensitivity, specificity, positive predictive value (PPV), negative predictive value (NPV), and accuracy of gray-scale US features, CDFI pattern, VTI score, and SWV value in differentiating malignant from benign thyroid nodules were compared with chi-square statistics. The diagnostic performance of gray-scale US features, CDFI pattern, VTI imaging score, and SWV value in the differential diagnosis between benign and malignant thyroid nodule were all assessed using receiver operating characteristic (ROC) curve analysis. Areas under the ROC curve (Az) and the 95% confidence intervals (CIs) of the Az values were calculated and compared using the* z* test. The ROC curve represents sensitivity versus 1-specificity for all possible cut-off values for prediction of malignancy. Cut-off values for SWV for the diagnosis of malignant thyroid nodules were defined using Youden's index. Multivariate logistic regression analysis was used to assess independent factors in predicting PTMCs. Odds ratios (OR) with relative 95% CIs were calculated to determine the relevance of all potential predictors of PTMCs. Statistical significance was accepted as *P* < 0.05.

## 3. Results

### 3.1. Pathologic Diagnoses

In the 173 thyroid nodules, there were 96 (55.5%) malignant nodules and 77 (44.5%) benign nodules. All the 96 malignant nodules were found to be papillary microcarcinomas after surgery. As to the 77 benign nodules, there were nodular goiter in 61 (79.2%), follicular adenoma in 1 (1.3%), and Hashimoto nodule caused by Hashimoto thyroiditis in 15 (19.5%), respectively.

### 3.2. Basic Characteristics and Conventional US Features Associated with Malignancy

Basic characteristics, gray-scale US features, and CDFI pattern of benign and malignant nodules were shown in the Table 1. Significant differences were found in age of patients, diameter of nodules, echogenicity of nodules, calcification of nodules, shape of nodules, halo sign, and vascularity pattern between benign and malignant nodules (all *P* < 0.05). Significant differences were found between any two types of vascular patterns. There was no significant difference in patient gender, solitary nodule or not, and location and margin between benign and malignant nodules (all *P* > 0.05).

### 3.3. ARFI Elastography Feature Associated with Malignancy

VTI scores of thyroid nodules with different pathology types were presented in Table 2. Score 1 was found in 2 nodules (1.2%), 1 nodule goiter, and 1 papillary carcinoma; score 2 in 44 nodules (25.4%), 19 papillary carcinomas, 23 nodule goiters, and 2 Hashimoto nodules; score 3 in 64 nodules (37.0%), 29 papillary carcinomas, 25 nodule goiters, and 10 Hashimoto nodules; score 4 in 43 nodules (24.9%), 30 papillary carcinomas, 10 nodular goiters, and 3 Hashimoto nodules; score 5 in 17 nodules (9.8%), 15 papillary carcinomas, and 2 nodular goiters; score 6 in 3 nodules (1.7%), 2 papillary carcinomas, and one follicular adenoma. The distribution of VTI score (score 1 to score 6) of benign nodules was significantly different from that of malignant ones. Most of benign nodules were classified to be score 1 to score 3 on VTI and most of malignant nodules were classified to be score 4 to score 6 (*χ* ^2^ = 16.5, *P* = 0.006). On the other hand, the distribution of VTI score of nodular goiters was not significantly different from that of Hashimoto nodules (*χ* ^2^ = 4.728, *P* = 0.316).

The mean SWV value of benign and malignant thyroid nodules was 2.57 ± 0.79 m/s (range: 0.90–4.92 m/s) and 3.88 ± 2.24 m/s (range: 1.49–9.00 m/s), respectively, and significant difference was found between them (*P* = 0.000).

### 3.4. Diagnostic Performance of Conventional US Features and ARFI Elastography

ROC curve was used to assess the diagnostic performance of gray-scale US feature, CDFI pattern, VTI score and SWV value in the differential diagnosis between benign and malignant thyroid nodules (Table 3). The Az values for hypoechogenicity, spot microcalcification, shape (taller than wide), absence of halo sign, type III vascularity, VTI score, SWV value were 0.610 (95-CI: 0.524, 0.696), 0.633 (95-CI: 0.550, 0.716), 0.686 (95-CI: 0.607, 0.766), 0.540 (95-CI: 0.453, 0.627), 0.455 (95-CI: 0.368, 0.542), 0.746 (95-CI: 0.672, 0.850), and 0.702 (95-CI: 0.625, 0.780), respectively. Az for VTI score was significantly higher than that for hypoechogenicity, absence of halo sign, and type III vascularity (*P* < 0.05). The optimal cut-off value with the highest sum of sensitivity and specificity (Youden cut-off) for VTI score and SWV in thyroid nodules was score 4 and 3.10 m/s. The corresponding sensitivity, specificity, accuracy, PPV, and NPV were presented in Table 3. Hypoechogenicity and absence of halo sign both had high sensitivities (94.8% and 99.0%) but the specificities were too low (27.3% and 9.1%). Among the conventional US features and ARFI elastography characteristics, VTI elastography score had relatively higher sensitivity, specificity, accuracy, PPV, and NPV.

### 3.5. Multivariate Logistic Regression Analysis for Predicting Malignant Nodules

After multivariate logistic regression analysis, gender, hypoechogenicity, shape (taller than wide), VTI score ≥ 4, and SWV > 3.10 m/s were found to be independent risk factors in predicting PTMC. After excluding the factors that had no predictive value on multivariate analysis, ORs of gender, hypoechogenicity, shape (taller than wide), VTI score ≥ 4, and SWV > 3.10 m/s were 3.591 (95% CI 1.116–11.558), 4.838 (95% CI 1.288–18.169), 5.478 (95% CI 2.161–13.887), 15.133 (95% CI 5.546–41.296), and 5.891 (95% CI 2.417–14.362), respectively (Table 4).

## 4. Discussion 

The treatment for PTMC, which is a tumor measuring less than 1 cm, is still a subject of controversy. PTMC had been considered as a “silent” cancer because most cases followed an indolent course with an excellent prognosis compared to the thyroid carcinoma > 1 cm [13]. Thyroid microcarcinoma is almost always of the papillary histotype and becomes evident at a relatively younger age and affects almost equally the two genders [14], as had also been demonstrated in this study. But high rate of multicentricity (32%–39.2%), bilaterality (20%–22.3%), invasiveness (18%–25%), lymph node metastases (16%–44.6%), and distant metastases (3%–7%) were reported in several series in nonincidental mPTC [15–17]. It is still necessary to make early and accurate diagnosis of PTMC.

The development of high-resolution US has resulted in a significant increase in the detection of nonpalpable small thyroid nodules, but the diagnostic value of US in nodules ≤1 cm was relatively lower than that in lesions >1 cm [18]. Contrast-enhanced ultrasound (CEUS) did not improve differential diagnosis between benign and malignant thyroid nodules [19]. US elastography is a newly developed dynamic technique that evaluates the degree of distortion of a tissue under the application of an external force and is based upon the principle that the softer parts of tissues deform easier than the harder parts under compression [20].

ARFI is a new technique of elasticity imaging that has been introduced into clinical practice in recent years. ARFI includes VTQ and VTI. VTQ allows quantification measurement of tissue elasticity in a selected region in the lesion whereas VTI estimates the elasticity of the whole lesion. Previous studies have shown that ARFI elastography seems to be a new tool for the differential diagnosis between benign and malignant thyroid nodules. VTQ can reflect the change of elasticity quantitatively in malignant nodules. The mean SWV value of malignant thyroid nodules was significantly higher than that of normal tissue or benign nodules [10, 12, 21–23]. In this study, similar results were found in nodules <1 cm. As the real-time elastography (RTE) displayed over the B-mode image in a color scale that ranges from red (for soft components) to blue (for hard components), VTI reflects the change of elasticity in a gray-scale image from bright (for soft components) to dark (for hard components). A VTI elastography image score standard had been developed in the previous study to evaluate the elasticity of lesion compared with the surrounding tissue [9]. The present study showed that VES 3 or less was highly suggestive of benign thyroid nodule whereas VES 4 or greater was highly suggestive of malignant thyroid nodule. However, there were no differences among other benign lesions. These results were similar to those in the nodules >1 cm. It can be concluded that the elasticity features of small lesions (size < 1 cm) were similar to that of large nodules (size > 1 cm).

**Figure 2:**
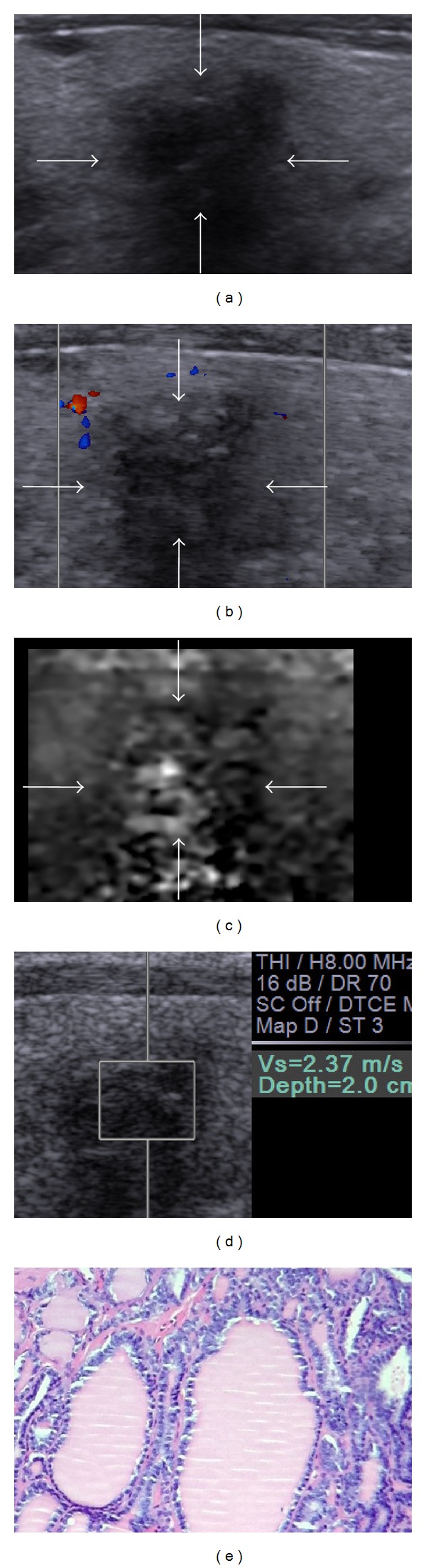
Images in a 54-year-old man with nodular goiter. (a) Conventional US shows marked hypoechogenicity, irregular shape, marked shadow, and ill-defined margin. (b) The color flow Doppler shows absent blood flow. (c) Score 2 is assigned at VTI. (d) The SWV of the nodule is “2.37” m/s. The conventional US features indicate probably malignant lesion; however, the ARFI characteristics show it probably benign. (e) Histology of the lesion confirms the diagnosis of nodular goiter. Hematoxylin and eosin stain, ×100.

**Figure 3:**
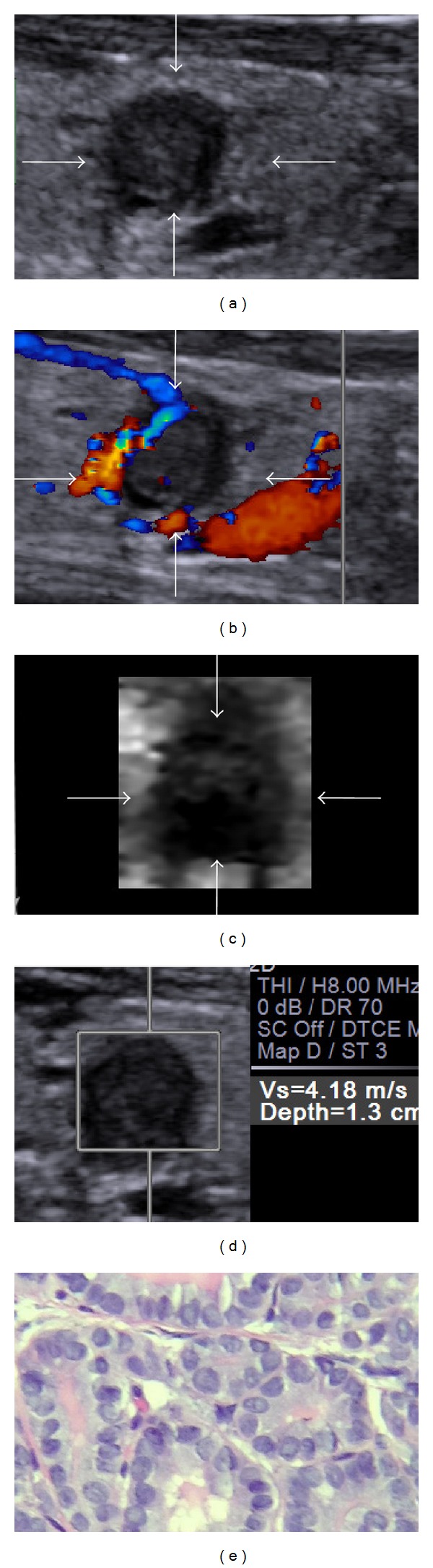
Images in a 39-year-old woman with papillary thyroid microcarcinoma. (a) Conventional US shows marked hypoechogenicity, a taller-than-wide shape, and well-defined margin. (b) The color flow Doppler shows perinodular blood flow. (c) Score 5 is assigned at VTI. (d) The SWV of the nodule is “4.18” m/s. The conventional US features indicate suspicious diagnosis; however, the ARFI characteristics help make a malignant diagnosis. (e) Histology of the lesion confirms the diagnosis of papillary thyroid carcinoma. Hematoxylin and eosin stain, ×400.

Several US features, including presence of calcification, hypoechogenicity, irregular margin, absence of a halo, predominantly solid composition, and intranodule vascularity, have been found to be associated with an increased risk of thyroid cancer (including cancers >1 cm and ≤1 cm) [24]. In this study, significant differences were found only in echogenicity of nodules, calcification of nodules, shape of nodules, halo sign being present or absent, and vascularity pattern between benign and malignant nodules. Furthermore, the differential diagnosis value of thyroid carcinoma based on these US features were compared to that based on ARFI characteristics. In previous studies, the sensitivity and specificity were 57–96.8% and 85–95.7% for VTQ, whereas 87% and 95.8% for VTI in the differential diagnosis of thyroid carcinoma [10, 12, 21–23]. The results of the present study showed that the Az values of ARFI characteristics (including “VTI elastography score > 4” and “VTQ SWV > 3.10 m/s”) were larger than Az values of US features; specifically, the Az value of “VTI elastography score > 4” was significantly larger than that of echogenicity and calcifications of nodules. In addition, VTI score had relatively higher sensitivity, specificity, accuracy, PPV, and NPV in all of these risk features. These results indicated that the differential diagnosis value of VTI for PTMC was better than that of conventional US features (Figures 2 and 3). In addition, the optimal cut-off value of VTI was found to be score 4 in PTMC, which was same to the previous study. The result showed that the VTI score standard could also be used in PTMC.

To find the independent risk factors in predicting malignant nodules, multivariate logistic regression analysis was done and gender, hypoechogenicity, shape (taller than wide), VTI score ≥ 4, and SWV > 3.10 m/s were found to be independent risk factors in predicting PTMC. The size of nodules, age of patients, single or multiple nodules, absence of halo sign, calcification, vascularity type were found to be of no value in predicting PTMC. The possible reason for why spot microcalcification was not a risk factor for PTMC may be that some PTMCs were in the early stage of carcinoma; thus, spot microcalcifications had not yet formed.

**Figure 4:**
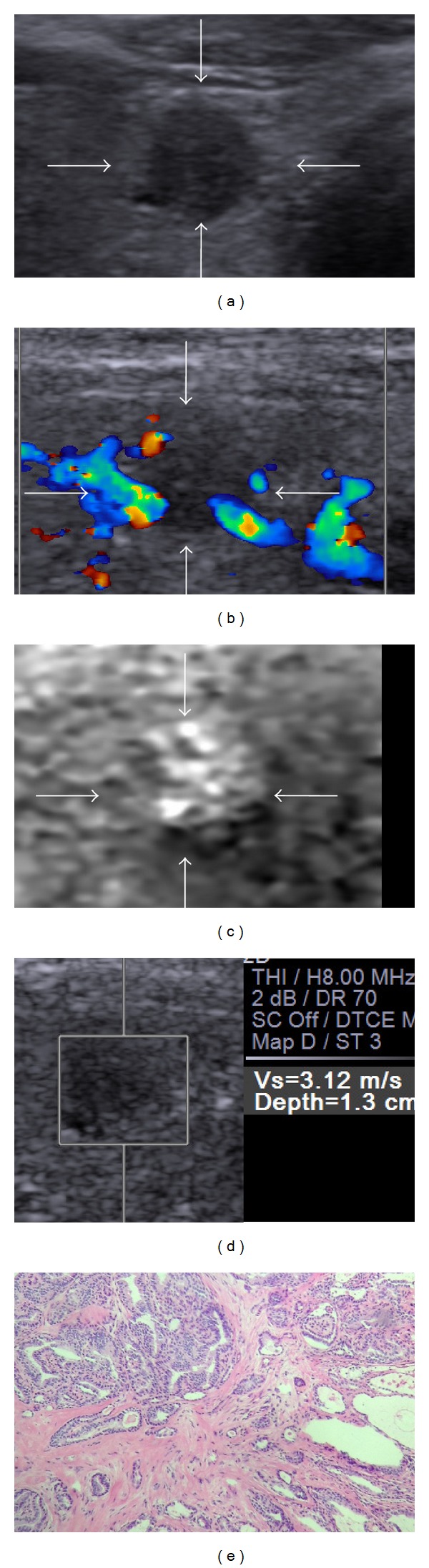
Images in a 62-year-old woman with papillary thyroid microcarcinoma. (a) Conventional US shows hypoechogenicity, a taller-than-wide shape, and well-defined margin. (b) The color flow Doppler shows absent blood flow. (c) Score 2 is assigned at VTI. (d) The SWV of the nodule is “3.12” m/s. The conventional US features and ARFI characteristics both indicate suspicious diagnosis. (e) Histology of the lesion confirms the diagnosis of papillary thyroid microcarcinoma. Hematoxylin and eosin stain, ×100.

Moon et al. [25] investigated the clinical implications of elastography as a prognostic factor in patients with PTMC or not. They found that a hard malignancy on the Rago score was significantly associated with pathologic extrathyroidal extension compared with a soft malignancy. The findings indicated that the elasticity of lesion might reflect the prognosis of PTMC. There were some PTMCs in the present study that were displayed as “soft” in VTI and VTQ, whether these “soft” PTMCs had relatively better prognosis than those “hard” PTMCs should be followed in the future (Figure 4).

The present study had some limitations. Firstly, cervical lymph node metastasis is related to higher incidence of recurrence; it is considered to be an aggressive clinical feature of PTMC [25, 26]. However, the main purpose of this study was to compare the diagnosis performances of conventional US with ARFI characteristics; thus, the study of lymph node metastasis was not carried out in the present paper. The relationship between cervical lymph node metastasis and US features will be one of the directions of future studies. Secondly, there are some technical concerns related to VTQ measurement in thyroid. The first is the limits of measurement. When the tissue was too soft or too hard, the SWV would be displayed as “x.xx m/s,” which made it difficult to get the exact value of SWV. In addition, the ROI of VTQ, which is 6 × 5 mm, cannot be altered. The diameter of some nodules in this study was 5 mm. When the ROI is placed on the nodule, some normal thyroid tissue would be included in the ROI; as a result, the SWV value might reflect not only the elasticity of nodules, but also some surrounding thyroid tissue. Thirdly, the population does not reflect a normal distribution of disease, whereas the suspicious nodules on conventional ultrasound. Therefore, the result of this study was only suitable under a specific clinic scenario and should be carefully interpreted in clinic practice. Finally, this study was based on a retrospective data, so some selection bias may exist. A large series study with a prospective design and long-term followup is needed.

In summary, VTQ can provide elasticity information of PTMC quantitatively and VTI can directly reflect the overall elastic property of thyroid nodule. VTI score 4 or greater and SWV value more than 3.10 m/s are highly suggestive of malignancy. Gender, hypoechogenicity, shape (taller than wide), VTI score ≥ 4, and SWV > 3.10 m/s are independent risk factors in predicting PTMC. ARFI elastography seems to be a valuable tool for the differential diagnosis between benign and malignant thyroid nodules ≤1 cm.

## Figures and Tables

**Table 1:** The basic characteristics and ultrasound features for the 157 patients with 173 thyroid nodules.

Characteristics	Benign	Malignant	*P*
Patients (*n* = 157)			
Sex (Male/Female)	20/51	15/71	0.108
Age (yrs)	54 ± 8 (32–71)	49 ± 11 (22–78)	0.001
Single nodule/multiple nodules	11/60	20/66	0.224
Nodules (*n* = 173)	*n* = 77	*n* = 96	
Diameter (mm)	8.2 ± 1.4 (5–10)	7.7 ± 1.5 (5–10)	0.031
Location			0.507
Left lobe	31	39	
Right lobe	44	51	
Isthmus	2	6	
Echogenicity			0.001∗
Markedly hypoechoic	20	46	
Hyperechoic	1	0	
Isoechoic	16	4	
Hypoechoic	36	45	
Mixed	4	1	
Calcifications			0.001∗
None	39	34	
Microcalification	22	53	
Macrocalcification	16	9	
Shape			<0.001∗
Ovoid to round	55	39	
Taller than wide	13	52	
Irregular	9	5	
Margin			0.176
Well-defined	48	50	
Ill-defined	29	46	
Halo sign			0.023
Present	7	1	
Absent	70	95	
Vascularity			<0.001∗
Type I	54	79	
Type II	14	0	
Type III	9	17	

Caption: *There were significant differences between benign and malignant nodules.

Significant differences were found in age of patients, diameter of nodules, echogenicity of nodules, calcification of nodules, shape of nodules, halo sign (all *P* < 0.05). Significant differences were found between any two types of vascular patterns (*P* < 0.001). There was no significant difference in gender, solitary nodule or not, location of nodules and margin of nodules between benign and malignant nodules (all *P* > 0.05).

**Table 2:** Pathology types and virtual tissue imaging scores of the thyroid nodules.

VTI Elastography Score	Benign nodules				Malignant nodules
	Nodule goiter (*n* = 61)	Follicular adenoma (*n* = 1)	Hashimoto thyroiditis (*n* = 15)	Total∗ (*n* = 77)	Papillary carcinoma (*n* = 96)
1 (*n* = 2)	1	0	0	1	1
2 (*n* = 44)	23	0	2	25	19
3 (*n* = 64)	25	0	10	35	29
4 (*n* = 43)	10	0	3	13	30
5 (*n* = 17)	2	0	0	2	15
6 (*n* = 3)	0	1	0	1	2

Caption: *In comparison with malignant nodules, *χ* ^2^ = 16.5,  *P* = 0.006 < 0.01.

The distribution of VTI score (score 1 to score 6) of benign nodules was significant different from that of the malignant ones. Most of benign nodules were classified to be score 1 to score 3 on VTI score and most of malignant nodules were classified to be score 4 to score 6 (*χ* ^2^ = 16.5, *P* = 0.006). But the distribution of VTI score of nodular goiters was not significantly different from that of Hashimoto nodules (*χ* ^2^ = 4.728, *P* = 0.316).

**Table 3:** Predictive value of conventional US features and ARFI in 173 thyroid lesions.

	BN (*n*)	CA (*n*)	Sensitivity (%)	Specificity (%)	PPV (%)	NPV (%)	Accuracy (%)	Az (95% CI)
US features								
Hypoechoic (including markedly hypoechoic)			94.8 (91/96)	27.3 (21/77)	61.9 (91/147)	80.8 (21/26)	64.7 (112/173)	0.610∗ (0.524, 0.696)
Yes	56	91						
No	21	5						
Spot microcalcification			55.2 (53/96)	71.4 (55/77)	70.7 (53/75)	56.1 (55/98)	62.4 (108/173)	0.633∗ (0.550, 0.716)
Yes	22	53						
No	55	43						
Shape (taller than wide)			54.2 (52/96)	83.1 (64/77)	80 (52/65)	59.3 (64/108)	67.1 (116/173)	0.686 (0.607, 0.766)
Yes	13	52						
No	64	44						
Halo sign			99.0 (95/96)	9.1 (7/77)	57.6 (95/165)	87.5 (7/8)	59.0 (102/173)	0.540∗ (0.453, 0.627)
Yes	7	1						
No	70	95						
Type III vascularity			17.7 (17/96)	88.3 (68/77)	65.4 (17/26)	46.2 (68/147)	49.1 (85/173)	0.455∗ (0.368, 0.542)
Yes	9	17						
No	68	79						
ARFI features								
VTI Elastography score > 4			61.4 (59/96)	88.3 (68/77)	86.8 (59/68)	64.8 (68/105)	73.4 (127173)	0.746 (0.672, 0.850)
Yes	9	59						
No	68	37						
VTQ SWV > 3.10 m/s			56.2 (54/96)	79.2 (61/77)	77.1 (54/70)	59.2 (61/103)	66.5 (115/173)	0.702 (0.625, 0.780)
Yes	16	54						
No	61	42						

Caption: BN, benign; CA, carcinoma; US, ultrasound; PPV, positive predictive value; NPV, negative predictive value.

*In comparison with “VTI elastography score > 4”, *P* < 0.05.

**Table 4:** Multivariate logistic regression analysis for predicting malignant nodules.

Characteristic	OR	95% CI	*P* value
Gender	3.591	1.116–11.558	0.032
Hypoechogenicity	4.838	1.288–18.169	0.020
Shape (taller than wide)	5.478	2.161–13.887	0.000
VTI score ≥ 4	15.133	5.546–41.296	0.000
VTQ SWV > 3.10 m/s	5.891	2.417–14.362	0.000

Caption: OR, odd ratio; CI, confidence interval

Gender, hypoechogenicity, shape (taller than wide), VTI elastography score ≥ 4, and SWV > 3.10 m/s were found to be independent risk factors in predicting PTMC.
